# Enhanced skeletal muscle contractile function and corticospinal excitability precede strength and architectural adaptations during lower-limb resistance training

**DOI:** 10.1007/s00421-023-05201-8

**Published:** 2023-04-25

**Authors:** Matthew T. Wilson, Angus M. Hunter, Malcolm Fairweather, Stewart Kerr, D. Lee Hamilton, Lewis J. Macgregor

**Affiliations:** 1 Physiology, Exercise, and Nutrition Research Group, Faculty of Health Sciences and Sport, University of Stirling, Stirling, UK; 2 Sportscotland Institute of Sport, Stirling, UK; 3 Life Fit Wellness, Healthcare & Exercise Centre, Falkirk, Scotland, UK; 4 Faculty of Health, School of Exercise and Nutrition Sciences, Institute for Physical Activity and Nutrition (IPAN), Deakin University, Geelong, Australia; 5 Department of Sports Sciences, School of Science and Technology, Nottingham Trent University, Nottingham, NG11 8NS UK

**Keywords:** Muscle adaptation, Strength, Resistance training, Tensiomyography, Contractile mechanics

## Abstract

**Purpose:**

Evolving investigative techniques are providing greater understanding about the early neuromuscular responses to resistance training among novice exercisers. The aim of this study was to investigate the time-course of changes in muscle contractile mechanics, architecture, neuromuscular, and strength adaptation during the first 6-weeks of lower-limb resistance training.

**Methods:**

Forty participants: 22 intervention (10 males/12 females; 173.48 ± 5.20 cm; 74.01 ± 13.13 kg) completed 6-week resistance training, and 18 control (10 males/8 females; 175.52 ± 7.64 cm; 70.92 ± 12.73 kg) performed no resistance training and maintained their habitual activity. Radial muscle displacement (Dm) assessed via tensiomyography, knee extension maximal voluntary contraction (MVC), voluntary activation (VA), corticospinal excitability and inhibition via transcranial magnetic stimulation, motor unit (MU) firing rate, and muscle thickness and pennation angle via ultrasonography were assessed before and after 2, 4, and 6-weeks of dynamic lower-limb resistance training or control.

**Results:**

After 2-weeks training, Dm reduced by 19–25% in the intervention group; this was before any changes in neural or morphological measures. After 4-weeks training, MVC increased by 15% along with corticospinal excitability by 16%; however, there was no change in VA, corticospinal inhibition, or MU firing rate. After 6-weeks training there was further MVC increase by 6% along with muscle thickness by 13–16% and pennation angle by 13–14%.

**Conclusion:**

Enhanced contractile properties and corticospinal excitability occurred before any muscle architecture, neural, and strength adaptation. Later increases in muscular strength can be accounted for by architectural adaptation.

## Introduction

Evidence suggests that initial training-induced increases in muscle strength are primarily the result of neural adaptation (Komi et al. 1978; Moritani and deVries 1979; Narici et al. 1989; Reeves et al. 2005). Early electromyography (EMG) studies demonstrated an association between increased strength and gross efferent output (Hakkinen and Komi 1983; Aagaard 2003). However, strength training adaptation varies greatly between individuals (Dankel et al. 2020; Roberts et al. 2018) with a cascade of neural, mechanical, and architectural adaptations taking place (Maughan et al. 1983; Higbie et al. 1996; Hubal et al. 2005; Erskine et al. 2010; Blazevich et al. 2008). Now, thanks to technological advancements, specific areas of the neuromuscular pathway can be investigated in isolation, shedding greater light on these initial adaptations. Specifically, individual motor unit (MU) properties (Van Cutsem et al. 1998; Vila-Chã et al. 2010), and parameters of the corticospinal tract and primary motor cortex (M1) (Carroll et al. 2002; Selvanayagam et al. 2011) have been studied. Collectively, these findings suggest that specific locations and responses underpin the early adaptations that typically occur within the first ~ 6 weeks after commencing strength training (Wilson et al. 2019). Observed responses include increased excitability (Goodwill et al. 2012; Weier et al. 2012) and decreased inhibition (Latella et al. 2012) of the corticospinal pathway, as assessed using transcranial magnetic stimulation (TMS) (Kidgell et al. 2017). Such alterations serve to increase motor neuron output and increase force production (i.e., strength) (Kidgell et al. 2017; Siddique et al. 2020). Although, these findings represent one of several areas of potential neural adaptation to resistance training they do not confirm alterations to MU behaviour. Therefore, further investigation, incorporating simultaneous assessment of different regions within the neuromuscular network would provide a more complete understanding of the neural contribution to strength adaptation.

Increased agonist muscle activation resulting from resistance training (Pucci et al. 2006; Jenkins et al. 2017) has been attributed to altered MU behaviour (Van Cutsem et al. 1998); specifically, increased firing rate following periods of resistance training has been detected by intramuscular EMG (Vila-Chã et al. 2010) and by decomposing surface EMG (dEMG) (Del Vecchio et al. 2019). However, conflicting findings have shown unaltered MU firing rate (Rich and Cafarelli 2000; Pucci et al. 2006; Sterczala et al. 2020), reaffirming some of the uncertainty around strength training adaptation. While EMG provides a useful, non-invasive method to assess MU adaptations, it does not allow us to make direct inferences about adaptations within the spinal excitatory/inhibitory networks. Therefore, concurrent measurements of MU discharge properties alongside the associated corticospinal networks of excitation and inhibition could provide clearer insight into the precise nature of the early adaptations to resistance training. To date, similar approaches have been performed in isolation, but as yet, simultaneous measurements across the breadth of the neuromuscular network have not been conducted as part of a robust training intervention paradigm.

We previously demonstrated that tensiomyography- (TMG) derived measurement of muscle belly radial displacement (Dm), which describes the magnitude of muscle deformation in response to percutaneous electrical stimulation, is inversely related to changes in muscle architecture (muscle thickness and fibre pennation angle) (Wilson et al. 2019). When assessing muscle atrophy during bed-rest, Šimunič et al. (2019) demonstrated an increase in Dm prior to any observable change in muscle thickness or pennation angle. This evidence suggests that the time-course of contractile mechanics and architectural changes are not exclusively linked. Although increases in pennation angle and muscle thickness have been shown to contribute to later increases in muscle strength (after > 4-weeks training) (Aagaard et al. 2001; Blazevich et al. 2003), it is not known whether contractile properties would be modified prior to these hypertrophy-orientated architectural adaptations. With early increases in strength primarily attributed to neural adaptations, it may be possible that early alterations in contractile properties are influenced by altered excitation–contraction (E–C) coupling (Calderón et al. 2014). Previously, TMG has been used to infer alterations in E–C coupling following exercise-induced muscle damage (EIMD) (Hunter et al. 2012); where increased contraction time (Tc), the time taken for the muscle belly to reach peak displacement, was associated with secondary EIMD markers. This research demonstrated the capability of TMG to assess early changes in contractile mechanics. To further our understanding of resistance training adaptation it would be beneficial to investigate the time-course of alterations in contractile mechanics in relation to other areas within the neuromuscular pathway. This exploration will provide improved clarity around the underpinning central and peripheral mechanisms responsible for strength adaptation. We anticipate that this research will expand on the growing body of work which challenges some long-held beliefs regarding the neural contributions to resistance training adaptation (Pearcey et al. 2021).

The aim of this study was to track the time-course of changes in muscle contractile properties with relation to muscle architecture and neuromuscular adaptation across a 6-week resistance training programme. By assessing the time-course of changes throughout the neuromuscular pathway we aimed to further elucidate the mechanisms responsible for increasing strength shortly after commencing a new programme of strength training. We hypothesised that over the course of the 6-week resistance training intervention increased strength would be accompanied by reduced Tc and Dm of agonist muscles, indicating improved contractile mechanics and that these contractile adaptations would occur prior to any discernible change to muscle architecture. We additionally hypothesised that increased strength during the initial stages of training would be accompanied by an increase in corticospinal excitability, reduced corticospinal inhibition, and increased MU firing rate.

## Methods

### Participants

A priori power analysis was conducted (G*power, version 3.1.9.4, Heinrich-Heine University, Dusseldorf, Germany) based on previously published data (increases in 1-RM back squat following 6-weeks resistance training in recreational athletes) (De Souza et al. 2018) and the following parameters: repeated measures analysis of variance (ANOVA), within-between interaction effect size = 0.2, power (1 − β) = 0.8 for the comparison between an experimental group and a control group. The study was not designed to compare within-group response differences between male and female participants. Power analysis showed that 18 participants per group were required to achieve an actual power of 0.81. In total, 43 eligible participants volunteered to take part in the study; 23 participants were randomly assigned to the training group and 20 participants to the control group with groups matched for height and body mass. A higher than indicated number of participants were recruited to account for potential dropouts, based on a similar previous training study (Wilson et al. 2019). Participants were unaccustomed to resistance exercise (i.e., novices) but were otherwise healthy and active. All participants met the UK national guidelines for aerobic physical activity of ≥ 75 min of vigorous activity per week; this was achieved by taking part in team sports 2 × per week, but their training did not involve any form of lower-limb resistance exercise for at least 6 months prior to involvement in the study. Participants were free from musculoskeletal injury or neuromuscular conditions. Prior to study commencement, participants provided informed consent, completed a physical activity readiness questionnaire and the screening questionnaire for TMS (Rossi et al. 2011); no volunteers were excluded for failing to meet the physical activity readiness requirements, one volunteer was excluded prior to testing for failing to pass the TMS screening. The study was approved by the University of Stirling NHS, Invasive or Clinical Research Committee, and all procedures were carried out in accordance with the ethical standards outlined in the latest revision of the Declaration of Helsinki.

### Experimental design

**Fig. 1:**
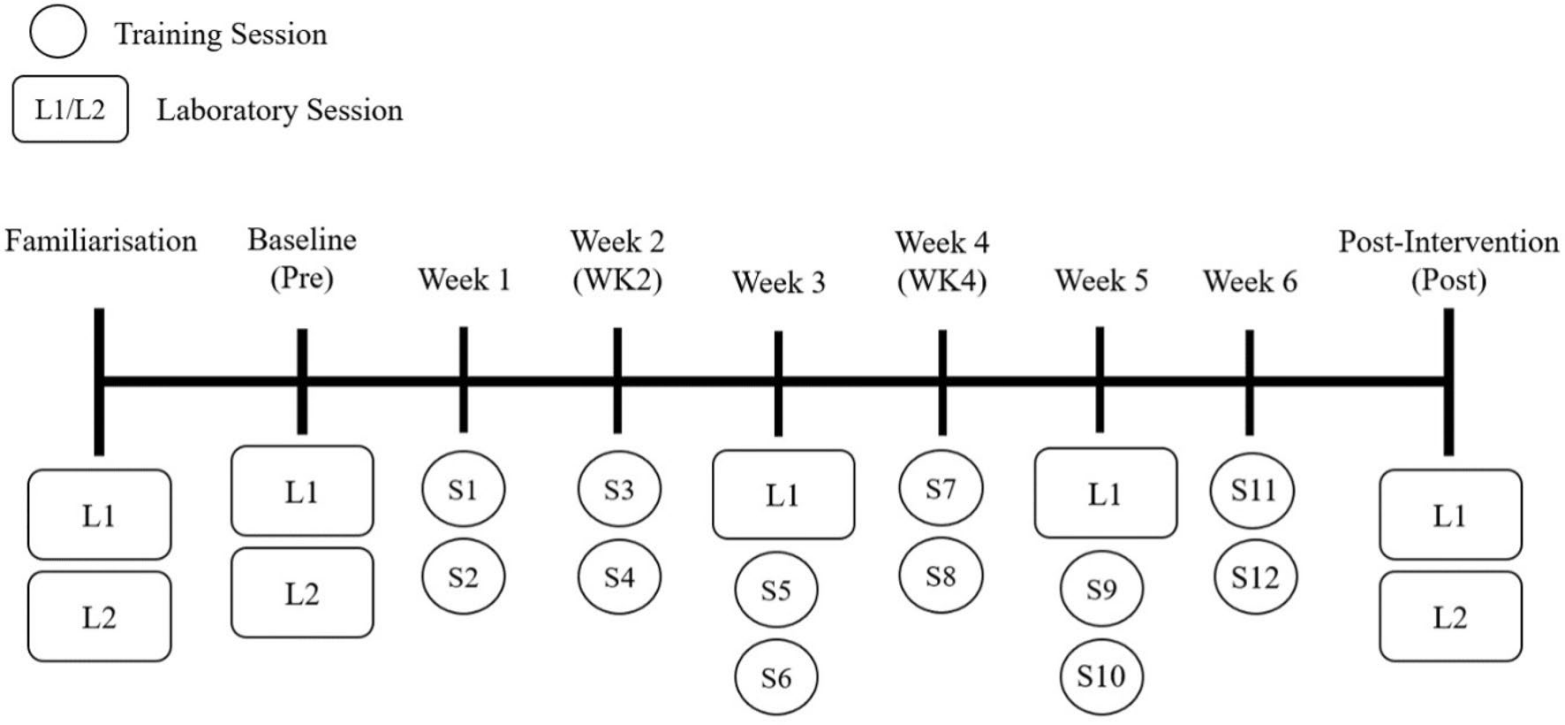
Schematic overview of the study design; L1—laboratory session 1; L2 – laboratory session 2; S1-12 – training sessions

A repeated measures design was used with a separate control group who were instructed to continue with their habitual physical activities and refrain from embarking upon a resistance training programme during the study. The study ran for 9 weeks in total (Fig. 1). Participants attended 2 laboratory sessions in week 1. The first of these laboratory sessions (L1) entailed: ultrasound imaging of muscle architecture, TMG assessment of contractile mechanics, isometric maximal voluntary contraction (MVC), corticospinal excitability/inhibition using TMS, maximal M-wave amplitude (M_max_) and percent of voluntary activation (VA), and dEMG during submaximal isometric contractions. After 48–72 h, the second laboratory session (L2) consisted of a 5-repetition maximum (5-RM) back squat (BS) test. Laboratory sessions carried out in week 1 served as familiarisation sessions, with the same schedule carried out a week later to obtain baseline measurements (Pre-). All measurements were performed by the same skilled investigator. Following Pre- measurements, the intervention group began 6-weeks of lower-limb resistance training (2 × per week) in addition to their habitual physical activity (Table 1). After the 2nd (Wk 2) and 4th (Wk 4) weeks of training, all participants from both groups underwent a repeat of session L1 to obtain time-point measures of any neuromuscular changes. Measurements taken at Wk 2 and Wk 4 were conducted at least 72 h after the previous training session to allow any intramuscular swelling or post-training fatigue in the intervention group to have recovered. Upon completion of the 6-week resistance training programme, all participants underwent repeats of sessions L1 and L2 (~ 4 days after the last training session) to obtain post-intervention measures (Post).

**Table 1:** Template of training programme used for the 6-week training intervention (week (1–6): number of sets × number of repetitions)

Session One		Session Two	
(1) Back squat (3-min rest)		(1) Back squat (3-min rest)	
1 & 2: 5 × 5–80% 5-RM		1–6: 3 × 10–70% 5-RM	
3 & 4: 5 × 5–82.5% 5-RM			
5 & 6: 5 × 5–85% 5-RM			
(2) Dumbbell walking lunges (2-min rest) (3) Dumbbell step-ups (2-min rest) (4) Goblet squats (2-min rest)		(2) Bulgarian unilateral split squats (2-min rest) (3) Barbell hip thrusts (2-min rest) (4) Box squats (2-min rest)	
1: 3 × 8	(RPE 6–8)	1: 3 × 8	(RPE 6–8)
2: 3 × 10	(RPE 6–8)	2: 3 × 10	(RPE 6–8)
3: 3 × 12	(RPE 6–8)	3: 3 × 12	(RPE 6–8)
4: 3 × 8	(RPE 7–9)	4: 3 × 8	(RPE 7–9)
5: 3 × 10	(RPE 7–9)	5: 3 × 10	(RPE 7–9)
6: 3 × 12	(RPE 7–9)	6: 3 × 12	(RPE 7–9)

Baseline 5-RM back squat was used to prescribe the loads for each participant, these were incrementally progressed from 80 to 82.5 to 85% of 5-RM during weekly Session One. Accessory exercise loads were progressed through a combination of rating of perceived exertion (RPE) and increased repetition number

*5-RM* 5-repetition maximum

### Skeletal muscle architecture and adipose tissue thickness

**Fig. 2:**
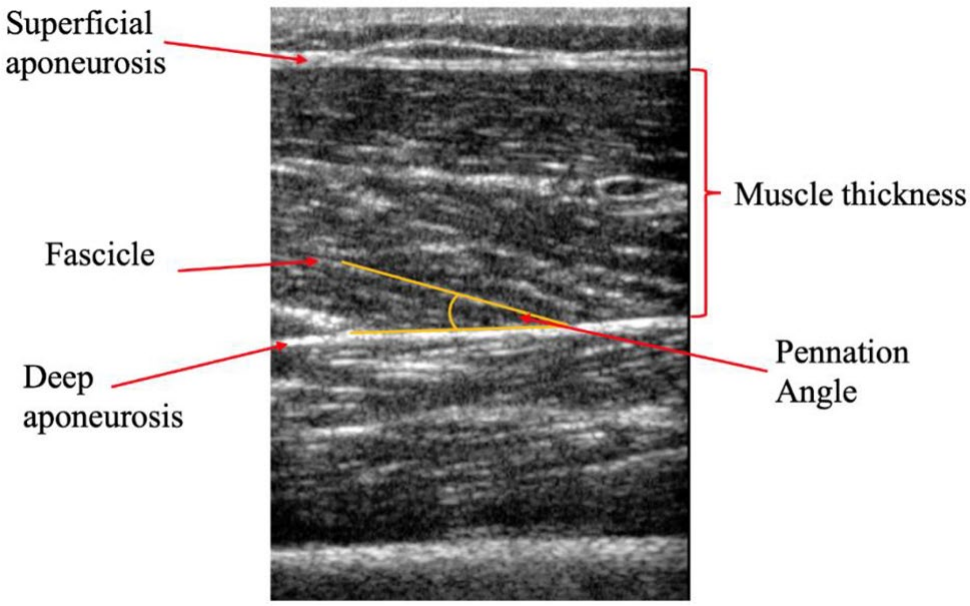
Representative longitudinal B-mode ultrasound image of the vastus lateralis. Muscle thickness is defined as the distance between the inside borders of the deep and superficial aponeurosis; muscle pennation angle is represented at the angle between the fascicle and the deep aponeurosis

Ultrasound images of vastus lateralis (VL) and rectus femoris (RF) muscle thickness and pennation angle were obtained from the dominant leg using a 12–4 MHz, 34.5 mm linear transducer (Philips Lumify, NV, USA). Muscle thickness was identified as the distance between the deep and superficial aponeurosis at the ends of each image; pennation angle was identified as the angle between a muscle fascicle and its deep aponeurosis (Fig. 2). Measurement sites of the VL and RF respectively were marked and recorded at 36% of the distance from the superior border of the patella to the anterior superior iliac spine and at 57% of the distance from the superior border of the patella to the anterior superior iliac spine (Wilson et al. 2019). Three longitudinal plane images were taken at each site and exported to Image J (National Institutes of health, Bethesda, MD, USA, version 1.8.0_112) for analysis. Analysis was performed unblinded by the same trained ultrasonographer that performed the scans. Mean values from the three images were used for analysis (Aagaard et al. 2001). Additionally, adipose tissue thickness was measured as the straight-line distance between the top of the facia and bottom of the cutaneous layer (Sterczala et al. 2020). Adipose tissue was measured to account for potential confounding variables which may have impacted EMG recordings throughout the study; adipose tissue is thought to act as a filter on EMG amplitude (Sterczala et al. 2020), and could inadvertently change EMG parameters between time-points.

### Tensiomyography

TMG assessment of contractile mechanics was carried out on the VL and RF using the same sites described above for ultrasound imaging, with positioning adjusted to ensure that the thickest part of the muscle belly was being measured (identified by manual palpation of the site). Participants remained in a supine position with the limb rested unfixed on a supportive pad which maintained a knee angle of 60° (0° = full knee extension). A digital TMG sensor (GK 40, Panoptik d.o.o., Ljubljana, Slovenia), with 1 μm sensitivity, was placed perpendicular to the skin surface, with two 5 × 5 cm self-adhesive electrodes (Axelgaard, USA) affixed equidistant on either side of the sensor with a 10 cm inter-electrode distance (centre to centre). The final position of electrodes and sensor were marked and measured with reference to the anterior superior iliac crest and superior border of the patella to allow accurate replication. A single 1 ms wide square wave stimulation (TMG S2, TMG-BMC Ltd., Ljubljana, Slovenia) was applied at an initial amplitude of 20 mA with a constant voltage of 30 V. Amplitude of stimuli was progressively increased by 10 mA, with an inter-stimulus interval of 10–15 s, until a plateau in the displacement–time curve was observed. From the peak twitch-time curve the following parameters were recorded from the TMG software (Version 3.6.16) and taken forward for analysis: maximal radial muscle displacement (Dm) and contraction time (Tc) during the linear portion of the ascending phase of the curve (10–90% of Dm).

### Maximal isometric voluntary contraction

Participants were seated in an isokinetic dynamometer with their dominant leg secured to a calibrated load cell (Biodex System 3, Medical Systems, New York, USA). Hip angle was 90° and knee angle was 60° flexion (0° = full knee extension) with the arm of the dynamometer being set such that the axis of rotation was in line with the participant’s lateral femoral epicondyle. Participants remained in this position for all subsequent testing procedures involving isometric contractions (TMS, M_max_, VA, dEMG). Participants were required to perform 3 MVCs of 5 s duration, in response to a randomly timed audio prompt. Participants were instructed to contract as hard and as fast as possible for the duration of the 5 s contraction and received consistent strong verbal encouragement throughout. A minimum of 60 s rest was permitted between MVC attempts. The maximum torque produced within the 3 attempts was used in analysis and for prescription of subsequent testing procedures. We calculated minimal clinically important difference (Lemieux et al. 2007) of strength adaptation from MVC by multiplying the intervention group pooled baseline standard deviation (SD) by 0.2, which corresponds to the smallest effect size. Therefore, our pooled baseline SD was ± 42.6 × 0.2 = 8.5 Nm.

### Transcranial magnetic stimulation

Single pulse TMS was used to elicit motor evoked potential (MEP) in RF, assessed using sEMG recordings (detailed below). Single 1 ms stimuli were applied over the contralateral M1 using a magnetic stimulator (Magstim 2002, The Magstim Company Ltd. Whitland, UK) with a 110 mm double cone coil attachment. Optimal coil location for MEP generation was determined by positioning the coil over M1, lateral to the vertex; coil location at which the largest MEP was elicited was identified and marked with semi-permanent ink (Goodall et al. 2009). This position was recorded for replication during subsequent visits. Quadriceps active motor threshold (aMT) was determined by increasing stimulator output in 5% increments, starting from 10%, until a discernible MEP was visible during sustained contraction at an intensity equivalent to 20% of participant MVC achieved during that testing session (Wilson et al. 1995); contraction intensity was maintained by the participant tracing their contraction force to a template visible on a computer monitor directly in front of them. Subsequent stimulations were delivered at 130% of aMT, this protocol has previously demonstrated a high level of reliability in our laboratories (Di Virgilio et al. 2022). A study by Temesi et al. (2014) found no differences between VL, RF, and vastus medialis for aMT or resting MT stimulus–response curves; suggesting that one muscle can be considered representative of all three.

To assess corticospinal excitability, participants contracted at 20% of MVC whilst 20 single pulse stimulations were delivered over M1, separated by 6 s. Corticospinal excitability was determined as the mean MEP peak-to-peak amplitude normalised to the maximal response elicited by motor nerve stimulation (%M_max_, as described below). To assess corticospinal inhibition participants contracted at 20% of MVC for 5 s duration whilst a single stimulation was delivered over M1, as previously reported by our group (Ntikas et al. 2021). This process was repeated 3 times with at least 60 s rest between contractions; mean cortical silent period (cSP) was recorded for analysis. Corticospinal inhibition was quantified as cSP duration, measured from stimulation artefact to the resumption of discernible, uninterrupted EMG activity, identified as an increase of ± 2 SD.

### Femoral nerve stimulation

Stimulation of the peripheral femoral nerve was conducted by attaching a self-adhesive surface electrode (cathode) lateral to the femoral artery, high over the femoral triangle, and anode over the gluteus maximus (Newell et al. 2021). Participants applied constant pressure to the cathode using a custom-built spring-loaded algometer to maintain adequate electrode–skin interface; participants were able to maintain consistent pressure on the cathode and reported no difficulties in applying pressure at rest or during contraction. Single stimuli were delivered while participants relaxed (DS7A, Digitimer Ltd., Hertforshire, United Kingdom), and stimulation intensity was incrementally increased until a plateau in twitch amplitude and RF M-wave (M_max_) were observed. The stimulation amplitude was then increased to 120% of the intensity that elicited M_max_ and participants received 3 further single stimulations whilst maintaining 20% of MVC to ensure M_max_ had been obtained. Next, a further pair of stimulations was delivered to assess VA via twitch interpolation (Merton 1954); first a superimposed stimulation was delivered during MVC (~ 2.5 s after onset of contraction, when maximum force had been attained), followed by a potentiated twitch stimulation 5 s after the superimposed stimulation. Maximum force was taken as the volitional force at the instantaneous delivery of stimulation (Zarkou et al. 2017); since this volitional force may not have always been maximum force level, the correction described by Strojnik and Komi (1998) was applied.

### Surface electromyography

Throughout TMS and femoral nerve stimulation protocols, sEMG was recorded from RF of the dominant leg, using a wireless system (Biopac Systems, Inc, Goleta, CA, USA). EMG was sampled at 2 kHz and filtered using 500 Hz low and 1.0 Hz high band filters. All signals were analysed off-line (Acqknowledge, v 3.9.1.6, Biopac Systems, Inc. Goleta, CA, USA). EMG activity was captured using a pair of Ag/AgCl surface electrodes (Ambu Ltd., UK) with inter-electrode distance of 2 cm. Recording site was shaved and abraded prior to electrode placement, as per surface electromyography for the non-invasive assessment of muscles (SENIEM) guidelines; which were also used to determine electrode location on the RF (50% distance on the line between anterior superior iliac spine to the superior border of the patella). A reference electrode was affixed over the patella of the dominant limb. Electrode positions were recorded in reference to anatomical landmarks for accurate replication in all subsequent testing sessions. Resting signals were inspected 80 ms prior to stimulation or voluntary contraction, to ensure the absence of artefact (Brownstein et al. 2017).

### Submaximal isometric trapezoid

Following the completion of TMS and peripheral nerve stimulation protocols, surface electrodes were removed, and the skin area cleaned. An electrode array sensor was fixed to VL to capture EMG for decomposition (as described below). Participants performed isometric knee extension following a trapezoidal target trace up to 60% of Pre-MVC. Participants were provided with a visual feedback trace throughout the task, linearly increasing and decreasing torque output at 10% of MVC/s either end of a sustained 10 s hold at 60% of MVC. If the participant failed to adhere to the visual feedback trace, they were allowed a second attempt following 2 min rest. Force equating to 60% of Pre-MVC was used in all subsequent testing sessions.

### Decomposition electromyography

A Delsys Trigno Lab wireless system was used to obtain four concurrent sEMG signals for decomposition. A 4-pin surface electrode array (Trigno-Galileo, Delsys, Natick) was affixed to the VL muscle belly, in the same location used for ultrasound imaging and TMG and secured with micropore tape. The reference electrode was affixed over the patella as per manufactures guidelines. VL was selected for measurement due to a typically lower subcutaneous adipose tissue thickness overlying this region compared to RF (reducing the spatial filtering influence of adipose tissue on EMG signals). All four channels of EMG were visually inspected, prior to data recording, to ensure excessive background noise and artefact were not present: baseline noise not exceeding 10 μV according to manufactures guidelines. Signal to noise ratio was calculated within the acquisition software (EMGworks4, Delsys, Natick) according to the following formula: 20log (S_RMS_/N_RMS_) [where S = EMG signal and N = baseline noise]. All EMG signals were low pass (fourth-order Butterworth, 24 dB/octave slope, 1750-Hz cut-off) and high pass (second-order Butterworth 12 dB/octave slope, 20-Hz cut-off) filtered prior to sampling at a rate of 20 kHz.

The four separate filtered EMG signals from the sensor array were decomposed into constituent MU action potential trains using Precision Decomposition III (PD III) algorithm (Neuromap, Delsys Inc, Boston, USA). The PD III technique was originally described by Adam and De Luca (2005), and subsequently refined by Nawab et al. (2010). PD III uses artificial intelligence to identify action potential shapes and assign them to individual MUs; with validity and reliability having been previously reported at various contraction intensities up to MVC (Hu et al. 2013). The accuracy of the dEMG process for each trapezoid contraction was assessed by reconstruct and test analysis (Nawab et al. 2010; De Luca and Contessa 2011; De Luca et al. 2015). This analysis assesses the level of firing rate accuracy of each identified MU and the number of errors per second, across the entire contraction (Accuracy = 1 − N_error_/N_truth_; where N_error_ is the total number of unmatched events, and N_truth_ is total number of true events). Only MUs which displayed > 90% accuracy were included for analysis. Mean firing rate (MFR) curves were smoothed a using 600 ms Hanning window, as recommended by the software manufacturer. From analysis of individual action potential trains, MFR during the constant torque contraction phase (60% of MVC) was calculated. MFR was calculated as the inverse of the averaging inter-spike interval during the distal 3 s of the constant torque phase. This 3 s period had been previously demonstrated in our laboratories to be the most reliable phase for analysis (Balshaw et al. 2017).

### 5-repetition maximum back squat

In the second lab session (L2), participants completed a 5-RM BS test. 5-RM exercise tests are reliable and valid for recreational athletes of trained and untrained backgrounds (Sascha and Künzell 2014). Following a standardised warm-up, participants completed a structured 5-RM protocol. Participants were permitted up to 3 attempts to obtain their 5-RM. Throughout the session participants reported rating of perceived exertion for each set completed, based on repetitions in reserved (RIR) (Zourdos et al. 2016). These RIR scores were used to guide incremental increases in load lifted for each exercise set. In week 1 (familiarisation), participants were assessed on correct and proper back-squat technique by qualified exercise professionals according to published exercise technical guidelines (Comfort et al. 2018). The 5-RM scores achieved in familiarisation were used to structure the incremental loads for Pre-intervention testing to ensure that 5-RM was achieved within 3 attempts and helped mitigate the learning effect before Pre-intervention measures were taken. Ankle eversion angle and stance width were recorded during baseline testing and replicated in post-intervention testing with the aid of marker tape (Schoenfeld et al. 2016). Participants did not have to complete all 3 attempts if they achieved 5-RM after 1 or 2 attempts. Participants were allowed 2-min rest intervals between sets 1–4, and 4 min between 5-RM attempts. The achieved Pre-intervention 5-RM load was incorporated into the post-intervention testing as the final warm-up set before the first post-intervention 5-RM attempt.

### Training intervention

The intervention group completed a 6-week resistance training programme (2 sessions per week, separated by ~ 72 h), consisting of compound lower-limb exercises following a linear periodisation model (Table 1). All training sessions were supervised by qualified instructors to ensure safe technique and correct performance of each exercise. Baseline 5-RM BS score was used to prescribe the loads and increments for each participant’s BS sets, such that participants worked at 80%, 82.5%, and 85% of 5-RM for Session One of Weeks 1–2, 3–4, and 5–6, respectively; training load in Session Two of each week was maintained at 70% of 5-RM for all 6 weeks. A rest period of 3 min was taken between each BS set. All other accessory exercise loads were guided by RIR to ensure sufficient training stimulus (Zourdos et al. 2016), such that training loads were adjusting to achieve a rating of perceived exertion of 6–8 in Weeks 1–3 and of 7–9 in Weeks 4–6, in addition the number of repetitions per set was increased from 8 to 10 to 12 throughout Weeks 1–3 and Weeks 4–6, respectively (Table 1). A rest period of 2 min was taken between each set of each accessory exercise. All participants were provided with drinks containing 20 g whey protein isolate dissolved in water, to aid recovery throughout the 6-week training period (West et al. 2017). Drinks were delivered at the end of each training session to the intervention group, and at the corresponding time points to the control group. To ensure compliance, each drink was provided directly by the investigatory team for both groups.

### Statistical analysis

All statistical analysis was carried out using Prism 8 (GraphPad software, CA, USA). Pre-, Wk 2, Wk 4, and Post-intervention data sets were checked for normality (Shapiro–Wilk test). Reliability was calculated from the control group for each dependant variable, using between-session test–retest intraclass correlation coefficient (ICC). For dEMG data, MU firing rate was analysed for each participant on a contraction-by-contraction basis; meaning data were not pooled from multiple contractions, nor multiple participants within a group. Two factor ANOVA with repeated measures was used to determine main effects of training intervention on muscle architecture, contractile mechanics, MVC, VA, corticospinal excitability and inhibition, and dEMG derived MU MFR [2 groups (independent factor) × 4 time-points (repeated measures factor)]. Where significant effect was detected Tukey post hoc analysis was used to identify where significant difference occurred. Cohen’s *d* effect sizes (ES) were calculated by; d = (Mean_1_-Mean_2_)/SD_pooled_, where SD_pooled_ = √[(SD_1_^2^ + SD_2_^2^)/2]. ES are interpreted as: ≤ 0.5 = trivial, 0.5–1.25 = small, 1.25–1.9 = medium, ≥ 2.0 = large, for untrained participants (Rhea 2004). All data are reported as mean and SD, with changes represented as percentage with upper and lower 95% confidence intervals (CI). Alpha was set at P < 0.05. Additionally, since the precise relationship between muscle contractile mechanics and muscle strength is not fully understood, where training-induced changes were observed, Pearson’s correlation coefficients (r) were determined post hoc to describe relationships between strength (MVC and 5-RM) and neuromuscular variables.

## Results

Three participants withdrew from the study; 1 from the training group for failing to complete > 90% of training sessions; 2 from the control group, respectively for sustaining an injury unrelated to the study and for failing to pass the TMS screening, leading to a final sample size of 40 participants; 22 in the training group (10 males/12 females; 173.48 ± 5.20 cm; 74.01 ± 13.13 kg; 19.14 ± 1.30 years) and 18 in the control group (10 males/8 females; 175.52 ± 7.64 cm; 70.92 ± 12.73 kg; 20.61 ± 0.94 years).

### Strength

Analysis of dynamic strength (5-RM) revealed a significant time effect [F_(1,38)_ = 104.1, P < 0.0001] across both groups, however this was overshadowed by significant Group × Test (4) interaction [F_(1,38)_ = 75.72, P < 0.0001]. The intervention group significantly increased their 5-RM BS strength (P < 0.0001, 95% CI 16.34 to 22.80), with no significant change in the control group (P = 0.53, 95% CI − 2.019 to 5.13). Post-intervention 5-RM BS strength was significantly greater (post hoc analysis) for the intervention group compared to control (P < 0.0001, 95% CI 17.27–43.59) (Table 2). Control group ICC = 0.88. Analysis of isometric strength (MVC) revealed a significant time effect [F_(2.54,96.42)_ = 15.01. P < 0.0001] across both groups, however a significant interaction effect [F_(3,114)_ = 9.297, P < 0.0001] showed the intervention group only significantly increased MVC by Wk 4 (P < 0.001, 95% CI − 42.41 to − 12.06) with further increase between Wk 4 and Post-intervention (P = 0.017, 95% CI − 25.30 to − 2.086). MVC was not significantly different between the intervention and control groups at Wk 4 (P = 0.92, 95% CI − 25 to 45.62) or at Post (P = 0.46, 95% CI − 17.0 to 62.0). Control group ICC = 0.94. Change in MVC from baseline was 27.2 Nm and 40.9 Nm at Wk 4 and Post respectively, thus exceeding the threshold of 8.5 Nm for clinical relevance. This provides confidence that the observed training adaptation substantially exceeds normal MVC variability. Level of VA did not change over time [F_(3,114)_ = 1.11, P = 0.348] nor was there a difference between groups [F_(3,114)_ = 2.60, P = 0.109] (Table 3). Control group ICC = 0.17.

**Table 2:** Time-course of % changes in strength measures across 6-weeks of lower-limb resistance training

	Knee extension MVC (Nm)				5-RM back squat (kg)	
	Pre	Week 2	Week 4	Post	Pre	Post
Intervention						
Mean ± SD	193.95 ± 42.67	207.66 ± 47.48	221.19 ± 44.15*	234.88 ± 53.50*#	60. 52 ± 20.71	84.24 ± 22.74*
∆ % from Pre [CI]		7.75% [– 29.95 to 2.54]	15.31% [– 42.41 to – 12.06]	21.81% [– 58.43 to – 23.43]	30.56% [– 22.80 to – 16.34]	
Control						
Mean ± SD	195.92 ± 41.77	200.5 ± 42.98	200.13 ± 41.83	201.20 ± 42.04	53.13 ± 12.40	54.61 ± 12.28
∆ % from Pre [CI]		2.70% [– 16.04 to 6.37]	2.64% [– 16.72 to 7.83]	3.11% [– 17.80 to 6.65]	3.03% [– 5.13 to 2.02]	

All data presented as mean ± SD

*CI* 95% confidence limits

*Significant change from Pre

^#^Significant change from Week 4, P < 0.05

**Table 3:** Voluntary activation (VA) for knee extensors; values are mean ± SD

Voluntary activation (%)				
	Pre	Week 2	Week 4	Post
Intervention	78.30 ± 14.83	69.68 ± 25.09	77.00 ± 21.13	72.69 ± 22.22
Control	83.47 ± 11.39	80.38 ± 16.45	80.67 ± 15.19	74.17 ± 23.42

### TMG-derived properties

**Fig. 3:**
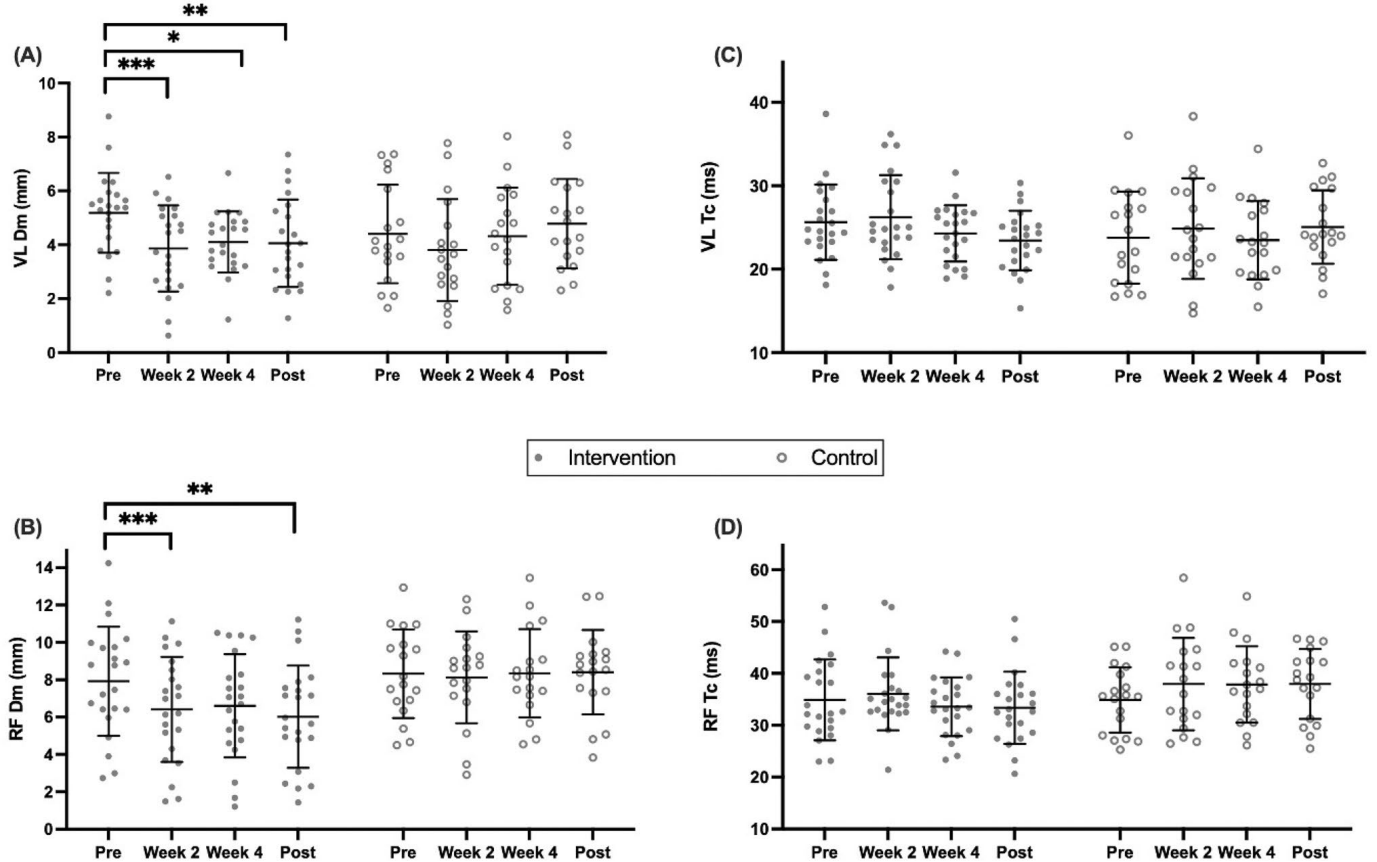
Time-course measures of contractile properties including: Dm (radial muscle displacement) in the vastus lateralis (VL) (**A**) and in the rectus femoris (RF) (**B**); Tc (contraction time) in the VL **(C)** and RF **(D)**, by group. Individual responses are shown in filled and outline circles, bars display mean ± SD; *significant decrease from pre, P < 0.05; **significant decrease from pre, P < 0.01; ***significant decrease from pre, P < 0.001

VL Dm showed a significant time effect [F_(2.68,101.7)_ = 6.17, P < 0.01] across both groups, however a significant interaction effect [F_(3,114)_ = 3.811, P = 0.01] revealed the intervention group only significantly reduced VL Dm by Wk 2 (P < 0.001, 95% CI 0.62–2.02). This reduction in VL Dm was maintained at Wk 4 (P = 0.02, 95% CI 0.11–2.06), and at Post (P = 0.01, 95% CI 0.26–2.01) (Fig. 3A). Control group ICC = 0.68.

Similarly for RF Dm, a significant time effect [F_(2.43,92.16)_ = 3.10, P = 0.04] was observed across both groups, however a significant [F_(3,114)_ = 3.08, P = 0.03] interaction effect showed the intervention group only significantly reduced RF Dm by Wk 2 (P = 0.003, 95% CI 0.47–2.55). This reduction was maintained at Post-intervention (P = 0.004, 95% CI 0.54–3.26), despite not being apparent at Wk 4 (P = 0.17, 95% CI − 0.39 to 3.03) (Fig. 3B). Control group ICC = 0.77.

Neither group significantly changed VL Tc over time [F_(2.37,90.01)_ = 2.17, P = 0.11], no significant interaction effect was found [F_(3,114)_ = 2.48, P = 0.07] (Fig. 3C). Control group ICC = 0.71. Similarly, neither group significantly changed RF Tc over time [F_(2.71,102.9)_ = 1.42, P = 0.24], nor was any significant interaction effect found [F_(3,114)_ = 2.17, P = 0.10] (Fig. 3D). Control group ICC = 0.61. There was no apparent relationship between VL Dm and MVC (r = − 0.102, P = 0.344) or 5-RM (r = − 0.267, P = 0.080), but a significant inverse relationship existed between RF Dm and MVC (r = − 0.245, P = 0.022) but not 5-RM (r = − 0.290, P = 0.057).

### Muscle architecture

Muscle architecture data is presented in Table 4. For VL muscle thickness, a significant time effect [F_(2.87,109.2)_ = 27.49, P < 0.0001] was observed across both groups, however a significant interaction effect [F_(3,114)_ = 14.62, P < 0.0001] revealed the intervention group only significantly increased VL muscle thickness by Wk 4 (P = 0.002, 95% CI − 0.24 to -0.05), and then further between Wk 4 and Post (P < 0.0001, 95% CI − 0.29 to − 0.13). Control group ICC = 0.91. Similarly, for RF muscle thickness, a main time effect [F_(2.70,102.60)_ = 15.31, P < 0.0001] was observed across both groups. However a significant interaction effect [F_(1,114)_ = 7.44, P < 0.001] revealed that the intervention group only significantly increased RF muscle thickness Pre-Post (P < 0.0001, 95% CI − 0.41 to − 0.20). Control group ICC = 0.83.

**Table 4:** *Muscle architecture measures* of the vastus lateralis (VL) and rectus femoris (RF) assessed over 6-week training programme

	Intervention Group						Control Group					
	Pennation angle (°)		Muscle thickness (cm)		Adiposity thickness (cm)		Pennation angle (°)		Muscle thickness (cm)		Adiposity thickness (cm)	
	VL	RF	VL	RF	VL	RF	VL	RF	VL	RF	VL	RF
Pre	19.04 ± 2.08	16.54 ± 1.91	2.29 ± 0.34	2.27 ± 0.37	0.65 ± 0.26	1.10 ± 0.46	18.74 ± 2.55	16.02 ± 2.97	2.18 ± 0.41	2.16 ± 0.49	0.62 ± 0.30	0.99 ± 0.47
Week 2	19.01 ± 2.14	16.68 ± 1.80	2.34 ± 0.34	2.32 ± 0.37	0.61 ± 0.25	1.14 ± 0.47	18.97 ± 2.59	16.16 ± 2.69	2.23 ± 0.39	2.27 ± 0.46	0.62 ± 0.29	1.00 ± 0.41
Week 4	19.69 ± 2.15	16.99 ± 1.59	2.43 ± 0.39*	2.36 ± 0.36	0.64 ± 0.26	1.05 ± 0.44	18.93 ± 2.61	16.03 ± 2.42	2.27 ± 0.38	2.24 ± 0.29	0.60 ± 0.29	1.03 ± 0.42
Post	21.65 ± 1.98*	18.72 ± 2.07*	2.64 ± 0.41*#	2.58 ± 0.32*	0.65 ± 0.26	1.07 ± 0.49	18.97 ± 2.55	15.96 ± 2.47	2.25 ± 0.38	2.26 ± 0.41	0.64 ± 0.31	1.01 ± 0.48

Values are mean ± SD

*Significant increase from baseline

^#^Significant increase from week 4, P < 0.05

Whilst a time effect [F_(2.42,91.82)_ = 29.04, P < 0.0001] was observed across both groups for VL pennation angle, a significant interaction [F_(3,114)_ = 24.37, P < 0.0001] revealed that the intervention group only increased VL pennation angle Pre-Post (P < 0.0001, 95% CI − 3.46 to − 1.76). Control group ICC = 0.95. Similarly, for RF pennation angle, a time effect [F_(1.78,67.44)_ = 11.14, P < 0.001] was observed across both groups and a significant [F_(3,114)_ = 13.86, P < 0.0001] interaction revealed that the intervention group only significantly increased RF pennation angle Pre-Post (P < 0.001, 95% CI − 3.35 to − 0.99). Control group ICC = 0.92.

### TMS-derived properties

**Fig. 4:**
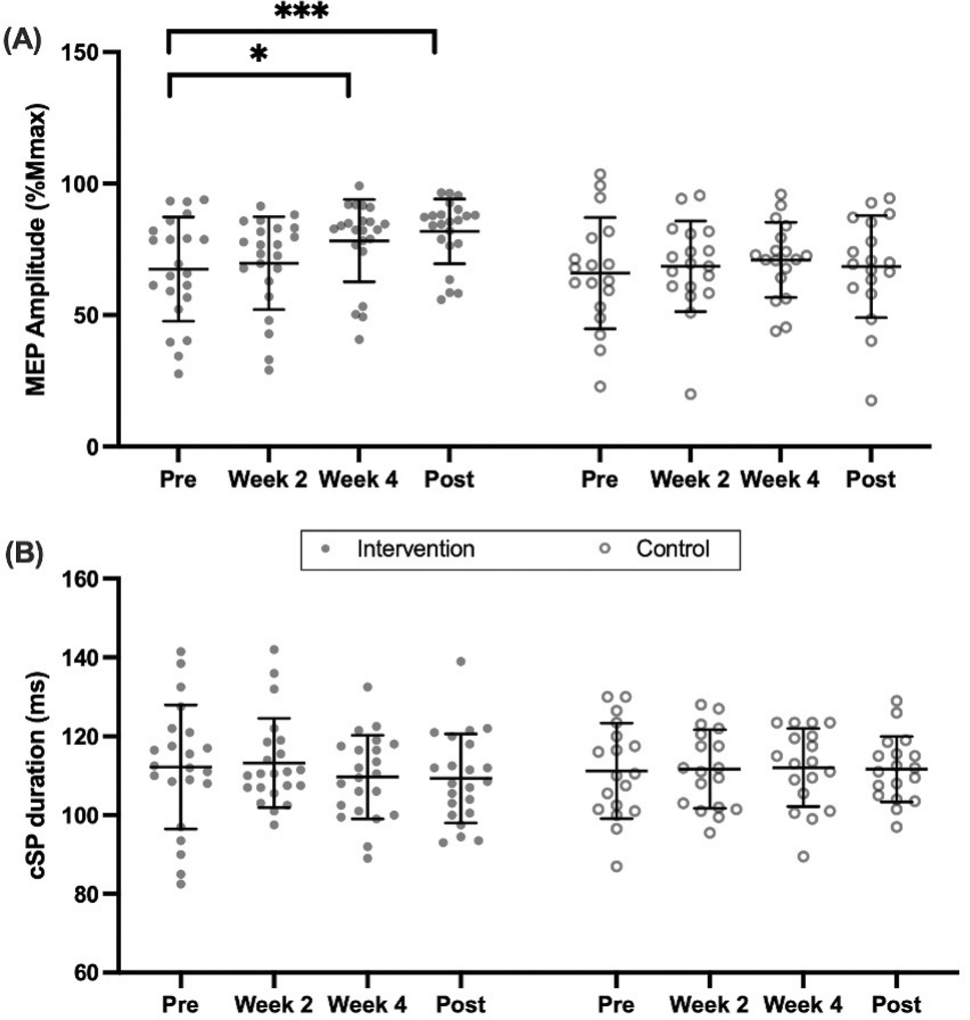
Time-course measures of corticospinal excitability (**A**) and corticospinal inhibition (**B**), assessed by Transcranial magnetic stimulation, by group. Individual responses are shown in filled and outline circles, bars display mean ± SD; *significant increase from pre, P < 0.05; **significant increase from pre, P < 0.01

**Fig. 5:**
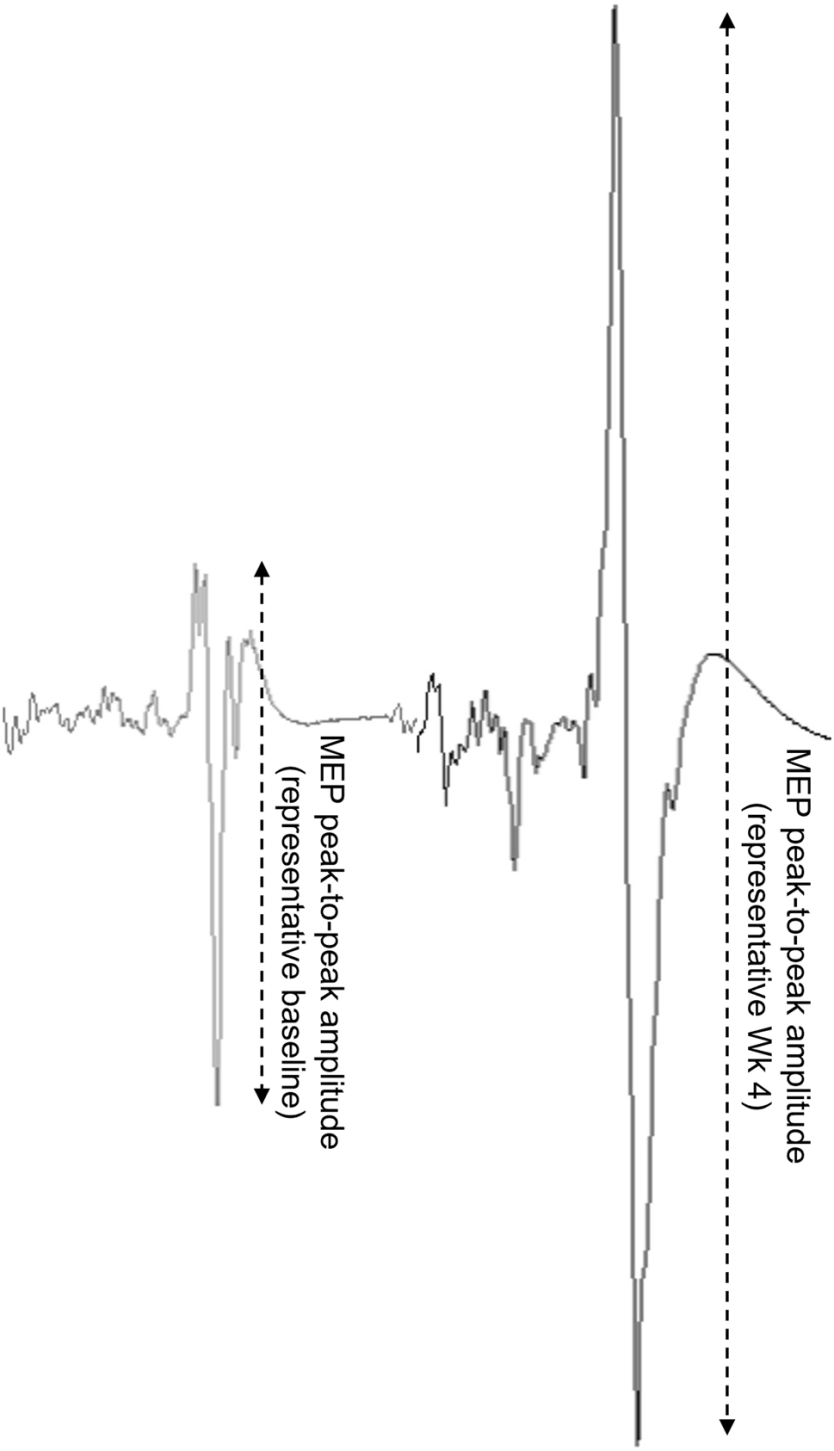
Representative motor evoked potential (MEP) amplitudes from an individual participant at baseline (left) and week 4 (right)

A time effect [F_(2.51,95.55)_ = 8,12, P < 0.001] was observed across both groups for corticospinal excitability (MEP amplitude), however, a significant interaction [F_(3,114)_ = 3.93, P = 0.01] revealed that the intervention group only significantly increased corticospinal excitability, by 16% at Wk 4 (P = 0.027, 95% CI − 20.66 to − 0.95); with this increase maintained Post-intervention (21%) (P < 0.001, 95% CI − 22.21 to − 6.53) (Fig. 4A). Representative MEPs at baseline and Wk 4 are presented in Fig. 5. Control group ICC = 0.74. MEP displayed a weak but significant correlation with MVC (r = 0.244, P = 0.022) and a stronger correlation with 5-RM (r = 0.410, p = 0.006).

Unlike corticospinal excitability, corticospinal inhibition (cSP duration) did not significantly [F_(2.63,99.93)_ = 0.49, P = 0.67] change over time and no significant [F_(3,114)_ = 0.69, P = 0.56] interaction effect was found (Fig. 4B). Control group ICC = 0.39.

### Motor unit firing rate

Despite the number of MUs identified at each time point being similar between groups (intervention—Pre, 15 ± 6; Wk 2, 16 ± 5; Wk 4, 15 ± 5; Post, 16 ± 4; control – Pre, 16 ± 5; Wk 2, 15 ± 6; Wk 4, 16 ± 5; Post, 16 ± 6), neither group showed a significant change in MU MFR over time [F_(2.49,94.78)_ = 2.70, P = 0.06], and no significant interaction [F_(3,114)_ = 1.89 P = 0.14] was found (Table 5). Control group ICC = 0.41.

**Table 5:** Mean firing rates (MFR) for vastus lateralis motor units assessed during 60% MVC isometric trapezoid contraction, across 6-week training programme

Mean firing rate (pps)				
	Pre	Week 2	Week 4	Post
Intervention	12.62 ± 2.48	14.21 ± 2.85	13.72 ± 1.88	14.19 ± 2.00
Control	12.83 ± 1.93	12.78 ± 2.71	13.53 ± 2.08	13.19 ± 2.01

Values are mean ± SD

*pps* pulses per second

## Discussion

In the present study we aimed to determine the time-course of neuromuscular adaptations to a resistance training programme among novice participants. Training took place over a 6-week dynamic resistance training period and participants demonstrated increased 5-RM BS supporting the effectiveness of the adopted training programme. A decrease in VL and RF Dm was observed after 2-weeks training and prior to any increase in isometric strength, neural adaptation, or change in muscle architecture. After 4-weeks of training, an increase in maximal quadriceps strength was observed, and accompanied by, increased corticospinal excitability; however, no changes in VA or MU MFR within the quadriceps were seen. Furthermore, an additional increase in maximal quadriceps strength was observed from Wk 4 to Post-intervention, alongside increases in muscle thickness and pennation angle. All effect sizes associated with major findings are presented in Table 6.

**Table 6:** Effect sizes associated with major findings (≤ 0.5 = trivial, 0.5–1.25 = small, 1.25–1.9 = medium, ≥ 2.0 = large)

	Interaction effect		Group effect
	Intervention	Control	
5-RM BS			
Pre-Post	0.90	0.13^*n.s*^	1.67
MVC			
Pre-Week 4	0.63		0.23^*n.s*^
Pre-Post	0.28		0.47^*n.s*^
VL Dm			
Pre-Week 2	0.86		
Pre-Week 4	0.82		
Pre-Post	0.73		
VL muscle thickness			
Pre-Week 4	0.39		
Pre-Post	0.52		
VL pennation angle			
Pre-Post	1.29		
RF Dm			
Pre-Week 2	0.53		
Pre-Week 4	0.46^*n.s*^		
Pre-Post	0.67		
RF muscle thickness			
Pre-Post	0.88		
FR pennation angle			
Pre-Post	1.09		
MEP amplitude			
Pre-Week 4	0.61		
Pre-Post	0.87		

*5-RM BS* 5-repetition maximum back squat, *MVC* maximal voluntary contraction, *VL* vastus lateralis, *RF* rectus femoris, *Dm* radial displacement; *MEP* motor evoked potential

^*n.s.*^no significant effect

Training-induced increases in 5-RM BS strength may be subject to a technique-learning effect; while any perceived strength gain based solely on specific adaptation to a repeatedly trained action should be considered with care, we caution against dismissing initial gains due to potential learning effect, as this effect could still be considered a relevant adaptation (Dankel et al. 2017). Nonetheless, increases in MVC, which we observed at Wk 4 and then again Post-intervention (Table 1), represent an unbiased measure of increased quadriceps strength. In this study, we observed increased quadriceps muscle contractile function and viscoelasticity of the muscle–tendon complex (Evetovich et al. 1997), as shown by decreased VL and RF Dm (Fig. 3A, B). Decreased quadriceps Dm before altered muscle architecture suggests a separate mechanistic change may be responsible for increased muscle tone. A recent study (Šimunič et al. 2019) demonstrated similar decreases in Dm prior to changes in muscle architecture during a recovery intervention following atrophy, also supporting the notion of mechanisms other than architectural adaptation being responsible for altered contractile properties. We previously reported that impaired E-C coupling has been represented by reduced Dm and increased Tc accompanying increased limb girth following EIMD (Hunter et al. 2012). However in the current study, no accompanying change in force output (Table 2) or Tc (Fig. 3C, D) was observed, indicating E-C coupling was not impaired. The present reduction in Dm was ~ 10% less than that shown by Hunter et al. (2012), indicating the less severe form of EIMD that commonly occurs in early stages of resistance training (Damas et al. 2015). A plausible explanation for reduced Dm is the alteration of intra-muscular tissue fluid content (Kasuga 2015) known to occur with EIMD in the early stages of resistance training (Chen et al. 2012). Such a non-invasive marker of contractile function could be useful for practitioners to gain objective insight into the efficacy of training interventions in their early stages. Future work should look to directly measure intra-muscular fluid content changes following resistance training concurrently with changes in contractile properties, to provide precise mechanistic understanding. High reliability of both Dm and Tc has been reported previously, using the same protocol that we have here adopted (Martín-Rodríguez et al. 2017) and the inter-individual spread in Dm recorded in this study is also similar to that reported elsewhere for lower-limb muscles (Llurda-Almuzara et al. 2020). Therefore, while we can be confident that the values at the upper and lower extremes of our range are not the result of measurement error, the significance of relatively high/low Dm within the context of our study is uncertain. This uncertainty arises because TMG measures skeletal muscle contractile mechanics in vivo, meaning that criterion referenced validity is inherently difficult to quantify (Macgregor et al. 2018). That we observed minimal relationships between changes in strength and Dm in both muscles suggests that the variance in Dm is not meaningful. TMG is not without limitations, we have previously raised questions around the external validity of the technique in relation to sports performance (Macgregor et al. 2018). While our current study, along with previous research (Wilson et al. 2019), has revealed associations between TMG-derived parameters and muscle function, it is still evident that the level of force evoked by electrical stimulation using TMG is < 10% of MVC (Ditroilo et al. 2011; Šimunič et al. 2011), which may—at least in part—explain the minimal relationships that we have observed between changes in Dm and strength.

Initial increases in muscular strength after 4-weeks of training were accompanied by neural adaptation in the form of increased MEP amplitude (Fig. 4A), partially confirming our secondary hypothesis. Increased MEP amplitude represents an increase in corticospinal excitability which includes the excitability of M1 and the efficiency of descending volley transmission through the spinal cord and into the muscles (Di Lazzaro et al. 2004). Similar results have previously been shown using dynamic and isometric training interventions, whereby increased corticospinal excitability has accounted for early strength gain (Griffin and Cafarelli 2007; Leung et al. 2013; Mason et al. 2017), although others have observed no change in MEP amplitude (Kidgell and Pearce 2010). Along with the findings of a recent meta-analysis (Siddique et al. 2020) the present study indicates that early-increases in muscular strength can be attributed to improved efficacy of neural transmission along the descending corticospinal tract. Interestingly, whilst corticospinal excitability remained increased (compared to baseline) at Post-intervention assessment, there was no further increase beyond Wk 4 (Fig. 4A). There are limited data regarding adaptations in corticospinal parameters after the initial increases presently observed as, by and large, previous studies have employed 3–5 week training interventions (Kidgell et al. 2017; Siddique et al. 2020). However, present data would suggest corticospinal excitability may not increase further after the initial observed change but rather, remain elevated after 4 weeks of training. We are not the first to report very early elevation in excitation, with little subsequent increase; Mason et al. (2020) reported greater area under the MEP recruitment curve following only one training session, with no further changes. We might speculate that the greater and more distal muscle mass involved in the present study (quadriceps vs. wrist flexors) may explain the delayed plateau in excitability response. Future work investigating the time-course of corticospinal excitability should look to determine the nature of this ‘ceiling effect’ and the influences of new training stimuli, including over a longer training duration to incorporate substantial muscle hypertrophy, and among a variety of muscle groups. The retention of the adaptations we have here observed could additionally be examined by prolonging measurements following cessation of the training stimulus (i.e., a detraining period). Since participants in this study were previously unaccustomed to resistance exercise, our findings are specific to novice exercisers. In this population we have here observed an early plateau in neuromuscular adaptation (i.e., within 6-weeks); whether a similar response could be expected among previously resistance trained individuals—for example, when starting a novel training programme or recommencing training after a period of interruption—is unclear. The principle of muscle memory associated with myonuclear domain size that has been reported in non-human mammalian muscle (Bruusgaard et al. 2012; Gundersen et al. 2018) has not been translated to humans (Psilander et al. 2019) but should not be dismissed as a possible mechanism for augmented adaptation to resistance exercise among non-novices. Therefore, future research should seek to build on our current findings by exploring early responses to resistance exercise in previously trained individuals.

At odds with recent meta-analyses (Kidgell et al. 2017; Siddique et al. 2020), and our own secondary hypothesis, was a lack in change of corticospinal inhibition (Fig. 4B). Recently, Ansdell et al. (2020) also demonstrated no change in cSP duration when assessing short-term training adaptations; however, these authors also saw no change in MEP amplitude unlike the present data. Therefore, it is possible the presently observed results are not due to alterations in M1 but rather somewhere else along the corticospinal tract. However, as TMS MEPs are unable to precisely differentiate between intra-cortical mechanisms (Brownstein et al. 2018) this cannot be confirmed. It should also be acknowledged that whilst shown to be a reliable method of analysis (Damron et al. 2008), cSP measurement does involve practitioner discretion as to when discernible EMG signal re-commences following the silent period; holding a potential for variance. Future work should explore techniques that allow such differentiation to be made within the corticospinal tract, such as stimulation of the cervico-medullary junction to determine efficacy of corticospinal-motor neuronal synapses (Nuzzo et al. 2016). Similarly, despite increases in voluntary strength (5-RM and MVC), we observed no change in VA. Trezise and Blazevich (2019) previously reported increased VA following a longer training programme (10 weeks) of similar frequency to our own, but it is noteworthy that this change showed no relationship with improved isometric strength. While VA may or may not be seen to increase following training, other neuromuscular adaptations are more clearly related to improving strength capability.

In order to gain comprehensive and concurrent insight into early-resistance training adaptations we employed sEMG decomposition to explore MU discharge property adaptations (Rich and Cafarelli 2000; Kamen and Knight 2004). No change in MU MFR, measured at 60% of MVC, was observed at any time point in either group (Table 5). The present MFR data is at odds with a recent study by Del Vecchio et al. (2019) who observed increased MFR in tibialis anterior after 4-weeks isometric training. Disparity between these findings may be due to methodological differences such as the training-specific tests in which MFR was assessed, and the specific equipment (and subsequent algorithms) used to collect MFR data. Another methodological difference was that Del Vecchio et al. (2019) pooled MFR data from multiple contractions, while we did not. Pooling MFR data from multiple contractions has the potential to alter numbers of low-threshold (faster firing) MUs (Van Cutsem et al. 1998) and inadvertently influence MFR. Indeed, MU MFR analysed on a per-contraction basis (as adopted here) suggests MFR is not altered Post-training (Beck et al. 2011; Stock and Thompson 2014; Sterczala et al. 2020). Furthermore, changes in adipose tissue thickness may arbitrarily alter MU properties derived from sEMG due to spatial filtering (Petrofsky 2008); to add a further measure of control to the present study, we measured adipose tissue thickness at the VL and RF electrode sites, with both remaining unchanged over the duration of testing (Table 4). It is possible that resistance training presently altered the recruitment thresholds of MUs, or the degree of MU hypertrophy (Sterczala et al. 2020); but this cannot be confirmed as it was not possible to measure relationships between MU property-recruitment thresholds. Therefore, in line with a previous suggestion (Contessa et al. 2016), future studies should look to these relationships when investigating training induced changes in MU behaviour to obtain greater clarity along the recruitment threshold spectrum.

Later increases in MVC strength (Wk 4 to Post) were accompanied by architectural adaptations in both VL and RF; namely increased pennation angle and muscle thickness (Table 2). Previously, increases in muscle thickness and pennation angle have been demonstrated following similar timeframes of resistance training (Blazevich et al. 2003; DeFreitas et al. 2011; Damas et al. 2015), and have been shown to be contributory to increased capacity for maximal force production (Aagaard et al. 2001; Campbell et al. 2013). It may be beneficial for future studies in this area to also include global measures of body composition to track changes in muscle mass. We observed that VL muscle thickness increased prior to RF (Table 2); this inter-muscle difference possibly resulting from differing stimuli during training (Floyd 2014). Previously, differences in hypertrophic response have been observed between VL and RF (Mangine et al. 2018), with differences in joint articulation involved in the exercises used in the present intervention suggested as explanatory; despite both muscles being controlled by the same innervation point (Page et al. 2019). Despite this inter-muscle difference in adaptation, our findings support the initial hypothesis of muscle architecture enhancements accounting for strength gain in the latter stage of training, as VL muscle thickness also increased from Wk 4 to Post-intervention. Interestingly, VL muscle thickness increased concurrently with corticospinal excitability after 4-weeks training, suggesting that neural and architectural adaptations may not be mutually exclusive. Additionally, corticospinal excitability remained elevated in the presence of further quadriceps architectural adaptation (Wk 4-Post), supporting the notion that a cumulative effect of neural and architectural adaptations account for strength gains. Despite our participants being unaccustomed to resistance exercise, all were healthy and otherwise physically active, as well as being young adults. We know that from the age of ~ 50 years, skeletal muscle mass is progressively lost at a rate of 1–2% per year (Baumgartner et al. 1998; Lauretani et al. 2003) and aged muscle is characterized by smaller muscle fibre diameter, more-varied fibre size, and reduced number of muscle fibres—specifically type II fibres (Frontera et al. 2000; McPhee et al. 2018; Tintignac et al. 2015). In older age, loss of muscle function is greater and more rapid than would be expected from the reduction in muscle mass alone, due in part to motor unit denervation and neuromuscular junction degeneration (McPhee et al. 2018; Mosole et al. 2014). Therefore, we caution against applying our present findings to older adult populations; similarly, further research is required to understand the early responses to novel resistance exercise among sedentary and/or unhealthy population groups.

As previously stated, our study was not designed to compare within-group response differences between male and female participants. Nonetheless, while the present sample (20 males/20 females) may be suitable to infer general conclusions regarding novice resistance exercisers, further research is needed to explore whether inter-sex differences may exist. It is well-understood that male and female responses to resistance exercise differ; men typically maintain ~ 10 kg greater muscle mass than women irrespective of overall body mass (Rossetti et al. 2017), so despite similar relative hypertrophy following resistance training (Abe et al. 2000; Hubal et al. 2005), in absolute terms men can gain up to twice as much muscle mass compared to women (Ivey et al. 2000). Inter-sex differences in TMG-derived properties have been less thoroughly investigated; among healthy individuals, women have been observed to present lower Dm than men in lumbar region (Lohr et al. 2020) and lower-limb musculature (Kusumoto et al. 2023). On the other hand, strong, resistance-trained women displayed greater Dm in lower-limb musculature compared to similarly well-trained men (Herring et al. 2021). Interestingly, Kojić et al. (2021) observed similar decreases in Dm among men and women following a 7-week resistance training intervention, but notably there were no inter-sex differences in Dm before training commenced among those individuals. Taken together, we might speculate that the existing evidence suggests the potential for TMG to distinguish between male and female musculature among healthy, non-resistance trained individuals, but the adaptive response to resistance training appears unlikely to differ between men and women.

## Conclusion

We are the first to demonstrate that increased quadriceps muscle contractile function and corticospinal excitability can be observed in the early stages of training prior to increased strength and architectural adaptation. However, precise mechanistic understanding of this change in skeletal muscle contractile mechanics remains to be determined. The integrated assessment approach employed in the present study supports the consensus that early strength is attributed to changes in the neural physiology, however further work is needed to precisely determine the location of adaptation. The cumulative effects of neural and physiological adaptations observed here may provide practitioners with some greater clarity of the time-course aspect of training-induced adaptations. This knowledge may inform the efficacy of training, rehabilitation monitoring and planning considerations.

## Data Availability

Data will be available upon reasonable request.

## Abbreviations

5-RM: 5-Repetition maximum
aMT: Active motor threshold
ANOVA: Analysis of variance
BS: Back squat
CI: Confidence interval
cSP: Cortical silent period
dEMG: Decomposition electromyography
Dm: Displacement
E-C coupling: Excitation-contraction coupling
EIMD: Exercise-induced muscle damage
EMG: Electromyography
ES: Effect size
ICC: Intraclass correlation coefficient
M1: Motor cortex
MEP: Motor evoked potential
MFR: Mean firing rate
M_max_: Maximal M-wave amplitude
MU: Motor unit
MVC: Maximal voluntary contraction
PD III: Precision Decomposition III
RF: Rectus femoris
RIR: Repetitions in reserve
SD: Standard deviation
Tc: Contraction time
TMG: Tensiomyography
TMS: Transcranial magnetic stimulation
VA: Voluntary activation
VL: Vastus lateralis
